# Breast Cancer Cell–Neutrophil Interactions Enhance Neutrophil Survival and Pro-Tumorigenic Activities

**DOI:** 10.3390/cancers12102884

**Published:** 2020-10-08

**Authors:** Lingyun Wu, Sugandha Saxena, Paran Goel, Dipakkumar R. Prajapati, Cheng Wang, Rakesh K. Singh

**Affiliations:** 1 Department of Pathology and Microbiology, University of Nebraska Medical Center, 985900 UNMC, Omaha, NE 68198-5900, USA; lingyun.wu@yale.edu (L.W.); sugandha.saxena@unmc.edu (S.S.); paran.goel@unmc.edu (P.G.); dipakkumar.prajapati@unmc.edu (D.R.P.); cheng.wang@unmc.edu (C.W.); 2 Vascular Biology and Therapeutics Program and Department of Pharmacology, Yale University, Yale School of Medicine, New Haven, CT 06520-8089, USA

**Keywords:** chemotherapy resistance, neutrophils, breast cancer, neutrophil extracellular traps, matrix metallopeptidase 9

## Abstract

**Simple Summary:**

Tumor cell–neutrophil interactions play an important role in tumor progression, metastasis, and overall survival. The purpose of this study was to examine the tumor cell–neutrophil survival and function. We observed that high neutrophil infiltration was associated with disease aggressiveness and therapy resistance, and breast cancer-derived factors significantly enhanced neutrophil survival, polarization, and expression of pro-inflammatory cytokines. The present study demonstrates the significance of tumor cell–neutrophil interaction in breast therapy resistance and neutrophils as a potential therapeutic target.

**Abstract:**

Breast cancer remains the most prevalent cancer in women with limited treatment options for patients suffering from therapy-resistance and metastatic disease. Neutrophils play an important role in breast cancer progression and metastasis. We examined the pro-tumorigenic nature of the breast cancer cell–neutrophil interactions and delineated the differences in neutrophil properties between the chemotherapy-resistant and the parent tumor microenvironment. Our data demonstrated that high neutrophil infiltration is associated with disease aggressiveness and therapy resistance. In the human breast cancer dataset, expression of neutrophil-related signature gene expression was higher in tumors from therapy-resistant patients than therapy-sensitive patients. We observed that breast cancer-derived factors significantly enhanced neutrophil survival, polarization, and pro-inflammatory cytokine expression. Breast cancer cell-derived supernatant treated neutrophils significantly expressed high levels of interleukin-1β (IL-1β), CC-chemokine ligand-2-4 (CCL2, CCL3, CCL4), inducible nitric oxide synthase (iNOS), and matrix metallopeptidase-9 (MMP9), and formed extracellular traps (NETs). Moreover, neutrophils showed increased secretion of MMP9 when cultured with the supernatant of chemotherapy-resistant Cl66-Doxorubicin (Cl66-Dox) and Cl66-Paclitaxel (Cl66-Pac) cells in comparison with the supernatant of Cl66-parent cells. Together, these data suggest an important role of breast cancer cell–neutrophil interactions in regulating pro-tumor characteristics in neutrophils and its modulation by therapy resistance.

## 1. Introduction

Breast cancer ranked as the second leading cause of cancer-related mortalities in the United States in 2020 [1]. The current therapeutic management for breast cancer patients includes surgery and chemotherapy drugs such as doxorubicin and paclitaxel [2]. Chemotherapy drugs target rapidly dividing cells with sensitivity to DNA synthesis or mitotic spindle interruption [3]. However, cancer cells can establish a resistance to these drugs, and various studies have reported a significantly lower survival rate of patients with chemotherapy resistance [4,5,6]. Therefore, there is an urgent need to delineate the precise mechanisms underlying chemotherapy resistance in tumors, thus establishing an optimized therapeutic plan for breast cancer.

The cancer cells can take advantage of multiple mechanisms to become resistant to chemotherapy drugs including upregulating inflammatory chemokine or cytokine production in tumor cells [7,8,9]. The upregulation of chemokines and cytokines can directly act on tumor cells or indirectly modulate the tumor microenvironment [10]. CXC-receptor-2 (CXCR2) and its ligands (CXCL1-3,5-8) are one such group of inflammatory chemokines considered pro-tumor factors in multiple cancer types [11,12,13]. Previously, our laboratory and other groups have also shown that targeting CXCR2 in cancer cells as well as in the host leads to enhanced chemotherapeutic response [8,9,14], inhibition of tumor growth, angiogenesis [15,16], and metastasis [17] in different cancer types indicating CXCR2 as an emerging target for cancer therapy [18,19].

One of the primary functions of CXCR2 and its ligands is to cause the recruitment of neutrophils, the hallmark of inflammation, through chemotactic responses [10]. The CXCR2 receptor, being present on neutrophils, responds to the upregulation of CXCR2 ligands in tumor sites, which results in higher recruitment of neutrophils into the tumor sites [10,20]. Recent reports have demonstrated the neutrophils’ pro-tumor role in the tumor microenvironment through the secretion of proteases such as matrix metalloproteinases (MMP) 9 and MMP2 as well as inflammatory factors including interleukin (IL)-1β [10,21], CC family ligands [10,22], and the formation of neutrophil extracellular traps (NETs) [10,23,24,25,26,27,28,29]. Gentles et al. demonstrated that higher polymorpho-nuclear cell (PMN) or neutrophil infiltration in tumors resulted in lower overall survival [30]. However, how cancer cells educate neutrophils toward a pro-tumor role remains unclear.

In our previous studies, we demonstrated the upregulation of CXCR2 and its ligands after chemotherapy treatment in breast cancer cell lines [14]. We observed higher metastasis [8] and a higher number of neutrophils in primary tumors and metastatic sites in tumors formed by the chemotherapy-resistant cell lines [31]. In this report, we hypothesize that neutrophil–tumor cell interactions play a pro-tumorigenic role in the breast cancer microenvironment. We analyzed the differences in pro-tumor factors, NETs formation, and neutrophil survival between the supernatant collected from the Doxorubicin-and Paclitaxel chemotherapy-resistant cells and parent cells. Our data demonstrated that high neutrophil infiltration is associated with disease aggressiveness and therapy resistance. We observed that breast cancer-derived factors significantly enhanced neutrophil survival, polarization, and expression of pro-inflammatory cytokines.

## 2. Results

### 2.1. A Higher Number of Neutrophils in Tumors from Breast Cancer Patients

**Figure 1:**
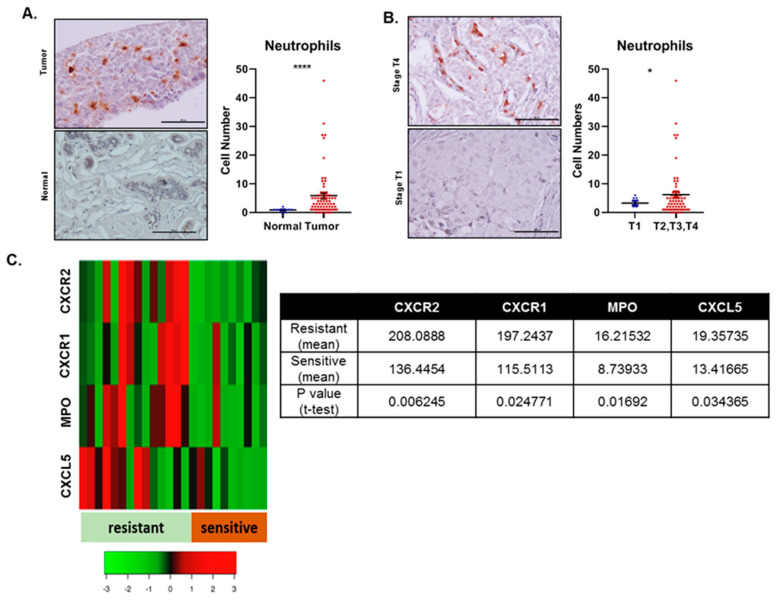
Neutrophils in human patients with different breast cancer stages and chemotherapy resistance. (**A**) Representative images and graphs showing higher neutrophil infiltration in the tumor tissues than normal tissues. (**B**) Representative images and graphs showing higher neutrophil infiltration in the tumors of T2, T3, and T4 stage patients (combined) compared to the tumors of T1 stage patients. The values are shown as number of neutrophils ± SEM, Unpaired t-test; * *p* < 0.05; **** for *p* ≤ 0.0001. (**C**) Heat map and table showing significantly higher levels of CXC-receptor-1 (*CXCR1*) CXC-receptor-2 (*CXCR2*), CXC-ligand-5 *CXCL5*, and Myeloperoxidase (*MPO*) in patients resistant to docetaxel chemotherapy. The values are shown as the mean and Student’s t-test.

We immunostained Myeloperoxidase (MPO), the marker for neutrophils in the tumor microenvironment, using the human breast cancer tissue array to understand the nature of the relationship between neutrophils and breast cancer. This array contains 80 cores with the TNM (tumor (T), node (N), and metastasis (M) ) stage and pathology grade along with healthy tissue. In this array, we observed that patients’ tumor cores had a significantly higher infiltration of neutrophils than the normal tissue (Figure 1A). Similarly, tumor cores of patients at relatively late stages (T2, T3, T4 stages) had a significantly higher infiltration of neutrophils into the tumor sites compared to the patients at the early stage (T1) (Figure 1B).

### 2.2. Breast Cancer Patients with Chemotherapy-Resistance Expressed Higher Levels of Neutrophil-Related Genes

Next, to understand the role of neutrophils to chemotherapy-resistance in breast cancer patients, we utilized the database GSE6434, which includes the information of twenty-four patients undergoing neoadjuvant docetaxel treatment. The patients were classified as chemoresistant and chemosensitive after the fourth cycle (12 weeks) of docetaxel. We analyzed the gene expression patterns of neutrophil associated gene such as MPO, neutrophil elastase-NE (ELANE), serine protease-CG, and genes involved in recruiting neutrophils to tumors such as CXCR1, CXCR2, CXCL1, CXCL2, CXCL3, CXCL5, CXCL7, CXCL8, and Interleukin-17 (IL17) in these two groups of patients. We observed significantly higher MPO levels, CXCR2, CXCR1, and CXCL5 in docetaxel-resistant patients than the docetaxel-sensitive patients. (Figure 1C, Table 1). We also observed higher levels of *IL17*, *NOS2*, *CXCL1-3*, *CXCL7-8*, *NE* (*ELANE*), and *CG* in resistant patients compared to chemotherapy-sensitive patients; however, this increase was not significant (Table 1). Higher levels of CXC-receptor and ligands in chemotherapy-resistant breast cancer patients suggest the recruitment of a higher number of neutrophils, characterized by the MPO gene, to the tumor sites of chemotherapy-resistant patients.

### 2.3. The Supernatant from Chemotherapy-Resistant Breast Cancer Cell Lines Enhanced Neutrophil Survival

**Figure 2:**
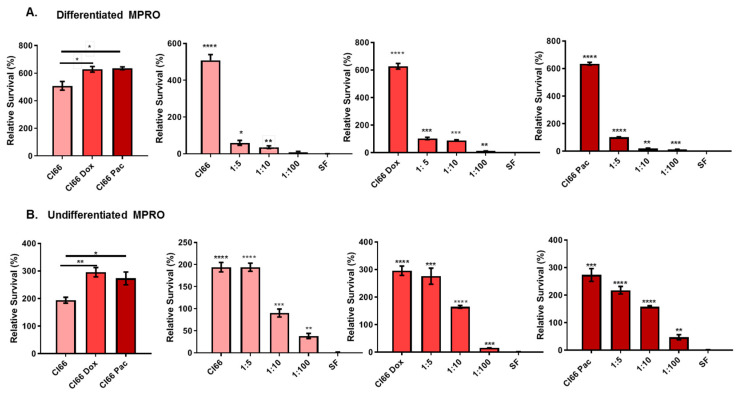
The chemotherapy-resistant cell lines Cl66-Doxorubicin (Cl66-Dox), and Cl66-Paclitaxel (Cl66-Pac). supernatants enhanced neutrophil survival. (**A**) Bar graph showing higher viability of differentiated MPRO in the supernatant of chemotherapy-resistant cell lines (Cl66-Dox and Cl66-Pac) in comparison with thee supernatant of parental Cl66 cells. Similarly, bar graphs showing an increase in the survival of differentiated neutrophils with an increase in the gradient of the supernatant of Cl66, Cl66-Dox, and Cl66-Pac cells. (**B**) Bar graph showing higher viability of undifferentiated MPRO in the supernatant of chemotherapy-resistant cell lines (Cl66-Dox and Cl66-Pac) in comparison with supernatant of parental Cl66 cells. Similarly, bar graphs show an increase in the survival of undifferentiated neutrophils with an increase in the gradient of the supernatant of Cl66, Cl66-Dox, and Cl66-Pac cells. The values are shown as mean ± SEM. The data are representative of three independent experiments performed in triplicate with similar results. Unpaired Student’s *t*-test; * *p* < 0.05; ** *p* < 0.01; *** for *p* ≤ 0.001; **** for *p* ≤ 0.0001.

We further investigated the effect of breast cancer cells on neutrophil survival. We cultured the differentiated and undifferentiated MPRO cells, a murine neutrophil cell line, in the supernatant of parent murine Cl66, Cl66-Doxorubicin (Cl66-Dox), and Cl66-Paclitaxel (Cl66-Pac). We observed that neutrophils cultured in the supernatant of breast cancer cells demonstrated significantly enhanced survival of both differentiated and undifferentiated MPRO cells (Figure 2A,B) compared with neutrophils cultured in the serum-free (SF) media. Additionally, this enhanced survival of neutrophils was significantly higher in the treatment of both differentiated and undifferentiated MPRO cells with the supernatant of chemotherapy-resistant breast cancer cell lines (Cl66-Dox and Cl66-Pac) in comparison with the parental cell-line Cl66 (Figure 2A,B). These results indicate that the neutrophils can survive longer in the breast tumor microenvironment, and this survival is further enhanced in chemotherapy-resistant tumors.

**Figure 3:**
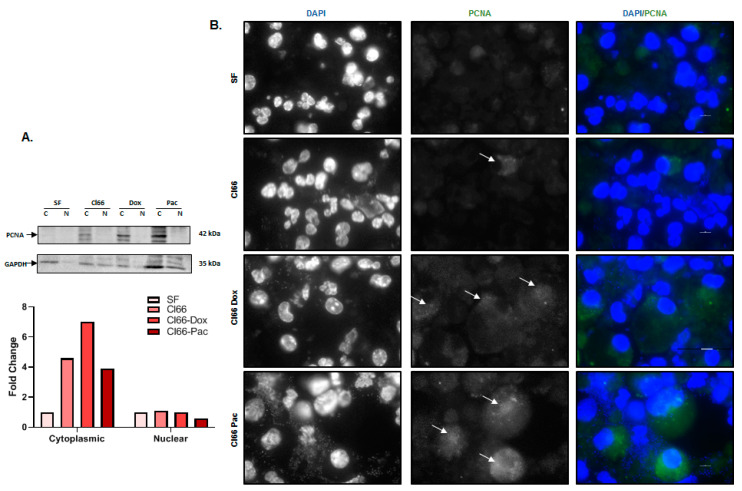
Breast cancer cell-derived factors enhance neutrophil survival by increasing cytoplasmic proliferating cell nuclear antigen (PCNA). (**A**) Western blot and a bar graph show a higher amount of PCNA in the cytoplasm of neutrophils treated with the supernatant of the Cl66, Cl66-Dox, and Cl66-Pac cell lines in comparison with the SF media. Blots were quantified using ImageJ software. Gaphd was used as a loading control and SF as a reference. the whole blot (uncropped blots) is shown in the Figure S1. (**B**) Immunofluorescence images showing a higher amount of PCNA in the cytoplasm of neutrophils treated with the supernatant of the Cl66, Cl66-Dox, and Cl66-Pac cell lines in comparison with SF media. PCNA was stained with the red nucleus as blue (DAPI). The data are representative of three independent experiments with similar results. The scale bar represents 100 μm.

Next, we investigated the underlying mechanism(s) for the enhanced neutrophil survival following treatment with breast cancer cells supernatants. Recently, proliferating cell nuclear antigen (PCNA) in the cytoplasm of neutrophils has been shown to play an important role in controlling neutrophil survival [32]. To evaluate whether PCNA plays a role in enhanced neutrophils’ enhanced survival, we cultured undifferentiated MPRO cells in the SF and supernatant of Cl66, Cl66-Dox, and Cl66-Pac cells and examined the PCNA protein levels. We observed more cytoplasmic-PCNA in the cytoplasm of neutrophils treated with breast cancer cell supernatants than those treated with SF media (Figure 3A). We further confirmed our observations using immunofluorescence and observed more cytoplasmic PCNA in neutrophils treated with the supernatant of the breast cancer cells than those treated with SF media (Figure 3B). These results collectively demonstrate the possibility of cytoplasmic PCNA being a significant player in breast cancer cell-induced neutrophil survival.

### 2.4. Breast Cancer Cells Modulated Expression of Neutrophil-Secreted Pro-Tumor Factors

**Figure 4:**
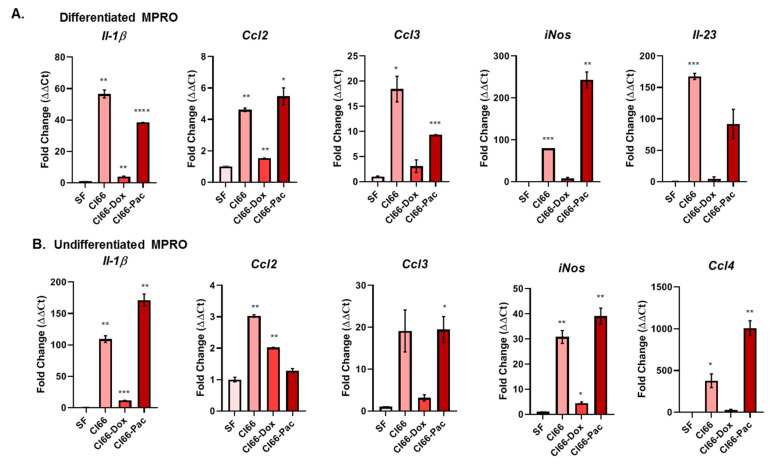
Breast cancer cells modulated the expression of neutrophil-secreted pro-tumor factors. (**A**) Bar graph showing fold changes in interleukin-1β (*Il-1β*), CC-chemokine ligand-2-4 (*Ccl2*, *Ccl3*, *Ccl4*), Interleukin-23 (Il23) and inducible nitric oxide synthase (*iNos*) expression in differentiated MPRO cells cultured in the supernatant of SF media, Cl66, Cl66-Dox, and Cl66-Pac cells using quantitative RT-PCR. The relative expression of *Gapdh* was used for normalization. MPRO cells cultured in the supernatant of SF media was treated as the control. (**B**) Bar graph showing fold changes in or the expression of *Il-1β*, *Ccl2*, *Ccl3*, *Ccl4*, and *iNos* in undifferentiated MPRO cells cultured in the supernatant of SF media, Cl66, Cl66-Dox, and Cl66-Pac cells using quantitative RT-PCR. The relative expression of Gapdh was used for normalization. MPRO cells cultured in the supernatant of SF media was treated as the control. The values are mean fold change ± SEM; and unpaired t-test with the assumption that both populations had the same SD; * *p* < 0.05; ** *p* < 0.01; *** *p* ≤ 0.001; **** *p* ≤ 0.0001. The data are representative of three independent experiments performed in duplicate with similar results.

We analyzed pro-tumor factors secreted by neutrophils on interaction with breast cancer cells. Both differentiated and undifferentiated MPRO cells, a murine neutrophil cell line, were cultured in the SF media, and the supernatant of parent Cl66, Cl66-Dox, and Cl66-Pac cell lines. The mRNA expression of different pro-tumor factors such as interleukin-1β (IL-1β), CC-chemokine ligand-2-4 (CCL2, CCL3, CCL4), Interleukin-23 (IL-23) and inducible nitric oxide synthase (iNOS), was analyzed. We observed higher levels of *Il-1β*, *Ccl2*, *Ccl3*, *Il23*, and *iNos* mRNA in the differentiated MPRO cells (Figure 4A) cultured in the supernatant of cancer cells compared with MPRO cells cultured in SF media. Similarly, the undifferentiated MPRO cells expressed higher levels of *Il-1β*, *Ccl2*, *Ccl3*, *Ccl4*, and *iNos* (Figure 4B) when cultured in the supernatant of cancer cells compared to SF media.

### 2.5. Neutrophils Treated with the Supernatant of Breast Cancer Cell Lines Formed NETs

**Figure 5:**
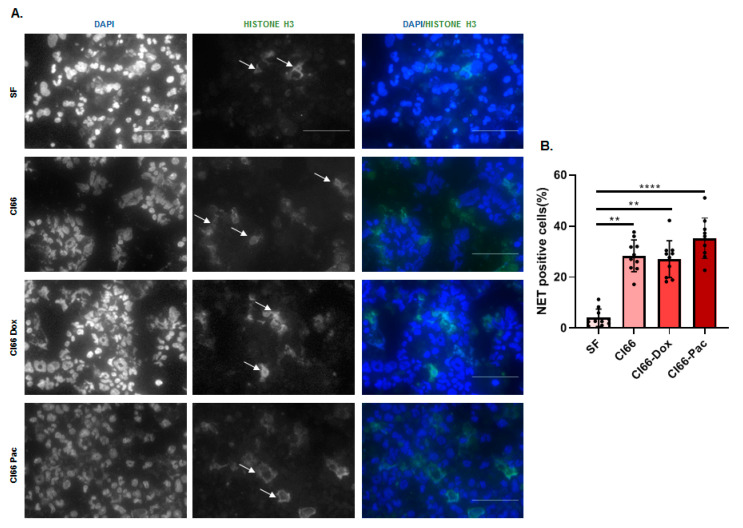
Breast cancer cells induce the formation of NETs. (**A**) Representative image showing the formation of NETs by MPRO cells in culture of the Cl66, Cl66-Dox, and Cl66-Pac cell lines in comparison with MPRO cells in SF media. The scale bar represents 100 μm. (**B**) A bar graph showing the percentage of NET-producing MPRO cells per field following treatment with supernatants from parental Cl66, Cl66-Dox, and Cl66-Pac cells. The data are a representative of two independent experiments with similar results. ** *p* < 0.01; **** *p* ≤ 0.0001.

Activated neutrophils in the tumor microenvironment can also create NETs, which can facilitate metastasis by capturing the circulating cancer cells [33]. They can assist in cancer progression through the proteases attached to the NETs such as matrix metallopeptidase-9 (MMP9) [10]. Next, we examined the effect of breast cancer cells on NET formation. We cultured MPRO cells in the SF media and the supernatant of Cl66, Cl66-Dox, and Cl66-Pac cells and examined them for NET formation by performing immunofluorescence. We observed that MPRO cells treated with the breast cancer cell–supernatant demonstrated the formation of NETs (Figure 5A,B), supporting evidence toward the pro-tumor nature of neutrophils in the breast tumor microenvironment.

### 2.6. Neutrophils Treated with the Supernatant of Chemotherapy-Resistant Cells Secreted MMPs

**Figure 6:**
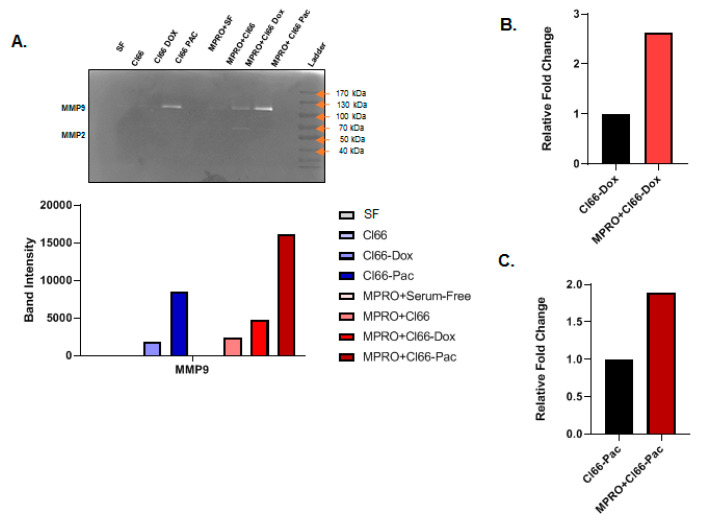
Neutrophils cultured in the supernatant of chemotherapy-resistant cell lines Cl66-Dox, and Cl66-Pac secreted higher matrix metallopeptidase-9 (MMP9). (**A**) Representative image of zymography and a bar graph showing MPRO cells cultured in the supernatant of the chemotherapy-resistant cell lines-Cl66-Dox and Cl66-Pac secreted higher MMP9 and matrix metallopeptidase-2 (MMP2). (**B**,**C**) Bar graph showing relative fold increase in the secretion of MMP9 by MPRO in culture with Cl66-Dox (**B**) and Cl66-Pac (**C**) in comparison with the supernatant alone of the Cl66-Dox and Cl66-Pac cell lines, respectively. The image was quantified using ImageJ software. The data are representative of two independent experiments with similar results.

Neutrophils are the major contributors of MMP9 in the tumor microenvironment [34] and contributors of MMP2 during chronic inflammation [35]. We treated differentiated MPRO cells in SF media as well as Cl66, Cl66-Dox, and Cl66-Pac supernatants, to test MPRO secreted MMP9 and MMP2 activity on gelatin zymogram. We also tested SF media and Cl66, Cl66-Dox, and Cl66-Pac supernatant for endogenous MMPs activity. We observed MMP9 activity in the supernatant of chemotherapy-resistant cell lines (Cl66-Dox and Cl66-Pac) and MPRO cells treated with supernatant of the Cl66, Cl66-Dox, and Cl66-Pac cell lines (Figure 6A). Moreover, MPRO cells showed enhanced MMP2 secretion on treatment with the Cl66-Dox supernatant (Figure 6A). It is important to note that although the supernatants of chemotherapy-resistant cell lines were positive for MMP9 activity, MPRO treated with the supernatant of these chemotherapy-resistant cell lines showed nearly two-fold higher MMP9-activity than the respective supernatants alone (Figure 6B,C). We observed the highest MMP9 activity in MPRO cells treated with supernatant of chemotherapy-resistant Cl66-Pac (sevenfold), followed by Cl66 Dox (twofold) in comparison with MPRO cells treated with the supernatant of the Cl66 cell lines (Figure 6A). These data suggest that breast cancer cell–neutrophil interaction can contribute to higher levels of MMPs, thus facilitating cancer progression and metastasis [36,37].

## 3. Discussion

Inflammation is a major hallmark of cancer [38], and both pro- and anti-tumorigenic properties of neutrophils [39] have been described. The data presented in this report strongly suggest that high neutrophil infiltration is associated with disease aggressiveness and therapy resistance. In the human breast cancer dataset, expression of neutrophil-related signature gene expression was higher in tumors from therapy-resistant patients than sensitive patients. We observed that breast cancer-derived factors significantly enhanced neutrophil survival, polarization, and expression of pro-inflammatory cytokines.

The role of context-dependent pro-tumorigenic neutrophils has been suggested previously for various human and murine tumor models [39]. Our group demonstrated higher levels of CXCR2 ligands in the chemotherapy-resistant breast cancer cells, both in vivo and in vitro [8]. The upregulation of such CXCR2 ligands results in higher neutrophil recruitment into the tumor sites [31]. In this report, we investigated the pro-tumorigenic nature of neutrophils recruited to the breast tumor microenvironment.

We observed more neutrophils in breast tumor tissue than healthy breast tissue and more neutrophils in late-stages (T2, T3, T4) in contrast to patients in the early-stage (T1). Our results indicates a positive association between neutrophils and human breast tumor progression. Next, we verified the relationship of neutrophils with chemotherapy-resistance in human breast cancer patients by utilizing a database comprising twenty-four females enrolled in the phase II study with neoadjuvant docetaxel (single agent). We found that patients with tumors resistant to the docetaxel expressed significantly higher levels of CXCR2, CXCR1, CXCL5, and MPO in comparison with patients with tumors sensitive to the drug. The higher neutrophil recruitment and chemokine expression in the chemotherapy-resistant tumors suggest a pro-chemotherapy-resistance role for neutrophils in human breast cancer cases. Our observation resonates with a study by Gentles et al. that indicated that higher polymorpho-nuclear cell (neutrophils) infiltration in tumors would lead to the lowest overall survival in cancer patients [19].

In this study, we report that the supernatant of chemotherapy-resistant cell lines (Cl66-Dox and Cl66-Pac) significantly enhanced the viability compared with the supernatant of the parental Cl66. We observed the differential impact of breast cancer cell-derived factors on the survival of undifferentiated and differentiated MPRO. Breast cancer cell-derived factors have a greater effect on undifferentiated MPRO because one of the factors produced by breast cancer cells is G-CSF, a potent molecule to pull out immature or undifferentiated neutrophils from the bone marrow. These immature cells are considered more pro-tumorigenic in comparison with mature differentiated neutrophils [31,40]. Mechanistically, we observed more cytoplasmic PCNA in neutrophils treated with the supernatant of cancer cell lines than SF media. Cytoplasmic PCNA in neutrophils can control their survival [32]. In addition, IL-1β is a vital neutrophil activator, and pro-survival cytokine [21] and the elevated expression of *IL-*1*β* can also be the reason for the prolonged survival time of neutrophils in the cancer supernatant.

To understand the nature of the interaction between breast cancer cells and neutrophils, we treated differentiated and undifferentiated neutrophils with the supernatant of parent and resistant cancer cells. We explored undifferentiated neutrophils because of their similarity to myeloid-derived suppressor cells (MDSCs). MDSCs are the heterogeneous populations of myeloid-derived cells, which are generally associated with immunosuppression in cancer cases [23]. We utilized two chemotherapy drugs, Doxorubicin and Paclitaxel, which have different mechanisms of killing tumor cells. Doxorubicin, an anthracycline, slows or stops the growth of cancer cells by blocking the Topoisomerase II enzyme needed for cell division and growth [41], and Paclitaxel is an antimicrotubule agent that attacks the cells during various phases of division. Microtubules play an essential role in the cell’s division and replication [42]. Thus, cancer cells will achieve resistance to survive these mechanisms by utilizing two independent pathways. We expected differential upregulation of tumor-promoting factors in the three different cell lines. Thus, to examine whether there are common tumor-promoting factors between parent and resistant cells that enhance neutrophil survival, we tested the expression of pro-inflammatory factors such as IL-1β, CCL2, CCL3, CCL4, IL23, and iNOS in neutrophils. The pro-inflammatory factors that we tested in our study were also pro-tumorigenic. IL1-β enhances neutrophil mobilization [27], survival [28], and the formation of NETs [27]. The CCL ligands can further recruit other immune cells including macrophages to the tumor microenvironment that facilitates cancer progression. High IL-23 and iNOS are related to breast cancer progression [43] and poor outcomes of platinum-based chemotherapy [44], respectively. However, differentiated neutrophils exclusively expressed *IL23*, whereas the undifferentiated neutrophils solely expressed CCL4 when cultured in the breast cancer supernatant. IL-1β is the only common factor, which was significantly upregulated in neutrophils by treatment with the supernatant of both parental Cl66 and resistant Cl66 cell lines in comparison with the SF control. Thus, undifferentiated and differentiated neutrophils have the potential to express pro-inflammatory factors in the tumor microenvironment differentially.

The formation of NETs or NETosis is another pro-cancer and pro-metastatic activity of neutrophils [45]. NETs or NETosis is a newly identified form of neutrophil cell death that has been shown to play a pivotal role in cancer progression and facilitating metastasis [10]. NETs can trap the circulating cancer cells [33], and proteases such as MMPs are attached to the neutrophil NETs, thereby facilitating metastasis [34]. Additionally, there is a recent report showing higher NET formation on treatment with different CXCR1/2 ligands [46]. Previously, we have seen that mice orthotopically injected with chemotherapy-resistant cell-lines in the breast showed higher metastasis in comparison with tumors formed by parental cell lines [8]. In this study, we analyzed the formation of NETs by neutrophils on treatment with the breast cancer cell supernatants. We observed the formation of NETs by the MPRO cells on treatment with the supernatant of the cancer cell lines. However, there was no difference in the NETosis on treatment with the supernatant of the parental and resistant cancer cell lines.

The neutrophil-released proteases in the tumor microenvironment can facilitate tumor metastasis through the degradation of the extracellular matrix [24,25], additionally, there is an association between increased levels of MMP9 and chemotherapy-resistance, which leads to lower survival rates [26]. In this study, we observed higher MMP9 activity in neutrophils treated with both Cl66-Pac (1.8 fold) and Cl66-Dox (2.5 fold) cell lines in comparison with the parental Cl66 cell line. Our result indicates a differential expression of MMP9 activity in neutrophils under the chemotherapy-resistant and parental tumor microenvironments.

Recent studies have provided evidence that tumor-associated neutrophils can be used as prognostic markers and regulate breast cancer metastasis [47,48,49]. Our current data demonstrate that breast cancer cells can influence neutrophils to facilitate cancer progression and metastasis through different complex mechanisms, and these mechanisms can differ between chemotherapy-resistant and parental breast tumor microenvironments. However, further studies are required on how cancer cells can influence different immune cells in the tumor microenvironment to improve the clinical outcome of immunotherapies 

## 4. Materials and Methods

### 4.1. Cell Lines and Reagents

We cultured the murine mammary adenocarcinoma cell line Cl66, Cl66-Dox, and Cl66-Pac [8] in Dulbecco’s Modified Eagle Media (DMEM: Mediatech, Hendon, VA, USA), 5% fetal bovine serum (FBS) (Atlanta Biologicals, Flower Branch, GA, USA), 1% L-glutamine (MediaTech), 1% vitamin solution (MediaTech), and 0.08% gentamycin (Invitrogen, Carlsbad, CA, USA) We added 500 nM doxorubicin (Bedford Laboratories, Bedford, OH, USA) and 400 nM of paclitaxel (Bedford Laboratories) in the medium of the resistant cell lines Cl66-Dox and Cl66-Pac, respectively. The detailed description, characterization, and the protocol used to establish the resistant cell lines are described in our previous studies [8,31].

The murine MPRO Cell Line, Clone 2.1(MPRO) (murine promyelocytes from ATCC, Manassas, VA, USA) were cultured in Iscove’s Modified Dulbecco’s Medium (IMDM, Sigma Aldrich, St. Louis, MO, USA) with 4 mM L-glutamine,10 ng/mL murine granulocyte-macrophage colony-stimulating factor (GM-CSF, Peprotech, Pittsburgh, PA, USA), and 20% heat-inactivated horse serum (Sigma Aldrich). The differentiation of MPRO was induced by 10 μM all-trans retinoic acid (ATRA, Peprotech) [50].

All cell lines were free of mycoplasma, as determined by the MycoAlert Plus Mycoplasma Detection Kit (Lonza, Rochester, NY, USA). For cell line authentication, Human DNA Identification Laboratory, University of Nebraska Medical Center, Omaha, NE, USA performed the short tandem repeat (STR) tests. Cell lines were maintained for six weeks at maximum stretch.

We plated an equal number of Cl66, Cl66-Dox, and Cl66-Pac cells in serum-containing DMEM media at 70% confluence of the dish. The next day, the cells were washed three times with HBSS (Sigma Aldrich), and 2 mL of SF DMEM was added to each well. Cell-free supernatant was collected after 24 h.

### 4.2. Human Breast Cancer Specimens

We purchased a human breast cancer tissue array, BR8015, from US Biomax (Derwood, MD, USA). The tissue array contained 50 cases of invasive ductal carcinoma, four ductal-lobular mixed carcinomas, eight invasive lobular carcinomas, eight medullary carcinomas, five adjacent healthy tissue, and five normal tissue. In total, the array contained 80 cases with a single-core per case including information about TNM and pathology grade.

### 4.3. Immunohistochemistry

Immunohistochemistry was performed as described previously [51]. In brief, we stained the human breast cancer array with MPO (Abcam, Cambridge, MA, USA) antibody. Immunoreactivity was determined using ABC reagent (Vector Laboratories, Burlingame, CA, USA) and DAB substrate (Vector Laboratories). Slides were counterstained with hematoxylin. The number of MPO positive cells was counted per core. The details of the antibodies are listed in Table 2. The representative pictures were acquired with a Nikon Eclipse E800 microscope (Nikon, Melville, NY, USA) and NIS-Elements BR 5.11.00 software (Nikon).

### 4.4. Bioinformatic Analysis

We analyzed the GSE6434 database [52] containing the gene expression profiles of twenty-four females with locally advanced breast cancer. The patients were enrolled in the phase II study and were undergoing the treatment of neoadjuvant docetaxel (single agent). The patients’ biopsies (primary cancers) were collected before chemotherapy treatment. The clinical response was assessed after the fourth cycle at 12 weeks. After 12-weeks of treatment using docetaxel, surgical specimens were also collected. For the detection of gene expressions on the patients’ biopsies, the Affymetrix U95Av2 GeneChip was utilized. We analyzed the gene expression patterns correlating with the response and de novo resistance to docetaxel from the initial pretreatment core biopsies. The heatmap was generated using Heatmapper [53].

### 4.5. mRNA Analysis

We examined the *Il-23*, *Il-1β*, *Ccl2*, *Ccl3*, *Ccl4*, *Inos*, and *Gapdh* expression in differentiated- (1 × 10^7^ cells) and undifferentiated-MPRO clone 2.1 cells (2 × 10^7^ cells). Both differentiated- and undifferentiated-MPRO clone 2.1 cells were treated with the supernatants of Cl66, Cl66-Dox, Cl66-Pac, and SF media cells for 24 h. Details of RNA isolation and reverse transcription are described in [54]. We prepared qRT-PCR reactions using PowerUp™ SYBR™ Green Master Mix (Thermo Fisher, Carlsbad, CA, USA), cDNA, gene-specific primers, and nuclease-free water. The results were analyzed using Thermo Fisher Connect (Thermo Fisher, Carlsbad, CA, USA). Mean Ct values of the target genes were normalized to mean Ct values of the endogenous control, *Gapdh*; [−∆Ct = Ct (GAPDH) − Ct (target gene)]. We calculated the ratio of mRNA expression of target genes versus *Gapdh* (2^(−∆Ct^) and further normalized it with the control (MPRO cells in SF) (2^(−∆∆Ct)^). Melting curve analysis was performed to check the specificity of the amplified products. The details of the sequence of gene-specific primers are in Table 1.

### 4.6. Immunofluorescence

We cultured the MPRO Clone 2.1 cells (1 × 10^6^ cells per well of a 96 well plate) in SF media and the supernatant of Cl66, Cl66-Dox, and Cl66-Pac for 4 h for staining NET using the Anti-Histone H3 (Abcam) antibody. For PCNA staining, MPRO Clone 2.1 cells (1 × 10^6^ cells per well of a 96 well plate) were treated with the supernatant of Cl66, Cl66-Dox, and Cl66-Pac and SF media for 24 h using the PCNA antibody (Cell Signaling, Danvers, MA, USA). From these treated cells, 100 L was cytospinned using Cytopro (Wescor) on glass slides. These slides were air-dried overnight. The cells on the air-dried glass slide were outlined using a Pap pen. Immunofluorescence was performed as described previously [55]. The representative pictures were acquired with a Nikon Eclipse E800 microscope (Nikon, Melville, NY, USA) and NIS-Elements BR 5.11.00 software (Nikon). For quantification of NET producing cells, we quantified the total number of nucleus per image, and the number of the nucleus in the vicinity of NETs was quantified as NET producing cells. The percentage was calculated using the formula: NET producing cells/Total number of the nucleus in the image × 100 = Percentage of NET producing cells.

### 4.7. Cell Viability Assay

Differentiated and undifferentiated MPRO Clone 2.1 cells (3 × 10^5^ per well in a 96-well plate) were treated with the supernatants of Cl66, Cl66-Dox, and Cl66-Pac and SF media. We treated the cells for 24 h with undiluted and diluted supernatant (1:5, 1:10, and 1: 100) and used SF DMEM as a control. WST Reagent (Sigma Aldrich, Milwaukee, WI, USA) was added to the cells for 4 h. The plate was read at a wavelength of 450 nm using an ELx800 (Bio-Tek, Winooski, VT, USA) plate reader.

### 4.8. Immunoblotting

MPRO Clone 2.1 cells (2 × 10^6^ in a 24-well plate) were treated with the supernatant of Cl66, Cl66-Dox, and Cl66-Pac and SF media for 24 h. Cells were lysed to collect the nuclear and cytoplasmic fraction as described by NE-PER Nuclear and Cytoplasmic Extraction Kit (Thermo Scientific, Rockford, IL, USA).

The protein concentration of extracted nuclear and cytoplasmic fraction of MPRO clone 2.1 cells treated with Cl66, Cl66-Dox, Cl66-Pac, and SF were determined using a Pierce™ BCA Protein Assay Kit (Thermo Scientific, Rockford, IL, USA). Protein samples (40 μg) were prepared using reducing 4× Laemmli buffer. The samples were electrophoresed on 12% sodium dodecyl sulfate (SDS) polyacrylamide gel. The electrophoresed protein samples were transferred to the Immobilon-p membrane (Millipore, Billerica, MA, USA). Membranes were blocked with 3% BSA (Sigma) in PBS for an hour at room temperature. Membranes were probed with specific primary antibodies overnight at 4 °C listed in Table 3. The following day, membranes were washed with tris-buffered saline containing 0.1% Tween 20 (TTBS) buffer three times and probed with respective secondary antibodies. Membranes were again washed thrice with TTBS buffer and visualized using the Luminata Forte Western HRP Substrate Kit (Millipore). We utilized NIH ImageJ Software Version 1.50i (National Institute of Health, Bathesda, MD, USA) or the quantification of immunoblots. The intensity of the bands of our protein interest was divided by the intensity of the band of their respective loading control. We also normalized the bands to the SF treated MPRO clone 2.1 cells.

### 4.9. Gelatin Zymography

The undifferentiated MPRO Clone 2.1 cells (1 × 10^7^ cells per 12-well) were treated with the supernatants of the Cl66, Cl66-Dox, and Cl66-Pac and SF media for 3 h. The cells were centrifuged and the cell-free supernatant was collected for gelatin zymography. The gelatin zymography was performed as described previously [56]. We utilized NIH ImageJ Software for the quantification of the zymography.

### 4.10. Statistical analysis

Analysis of the in vitro and in vivo data was performed using the Kruskal–Wallis one-way analysis of variance on ranks with Tukey’s test for multiple comparisons, and the Mann–Whitney U test or two-sample t-test for comparisons between two independent groups. We analyzed the results using GraphPad Prism 8.0 software (San Diego, CA, USA) and these were presented as mean ± SEM. A *p*-value ≤ 0.05 was considered statistically significant.

## 5. Conclusions

**Figure 7:**
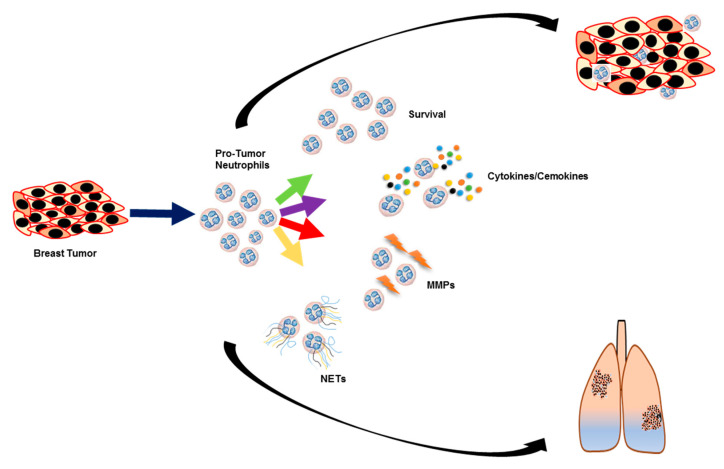
Breast cancer cells induce neutrophils to secrete pro-tumor factors, which aids tumor growth and metastasis. The pictorial diagram showing breast cancer cells enhances pro-tumor factors in neutrophils, which supports tumor growth and metastasis. Breast cancer cells increase the secretion of cytokines and chemokines as well as NET formation in neutrophils. In contrast, chemotherapy-resistant cells further enhanced neutrophil survival and secretion of MMP9, which can result in higher metastasis of chemotherapy-resistant cells.

Our current study is an initial attempt to understand how chemotherapy-resistant and parental breast cancer cells influence neutrophils. We demonstrate that breast cancer cell-derived factors can promote neutrophils to play a pro-tumor and pro-metastatic role through multiple mechanisms including upregulation of pro-tumor factors such as IL-1β, CCL2, CCL3, CCL4, IL23, and iNOS, NETosis, neutrophil survival longevity, and MMP9 secretion (Figure 7). Chemotherapy-resistant breast cancer cell-derived factors selectively enhance neutrophil survival and secretion of MMP9. These data suggest the role of tumor cell–neutrophil interaction in breast cancer progression and therapy resistance and neutrophils as a potential therapeutic target for the treatment of advanced-stage breast cancer patients.

## Figures and Tables

**Table 1:** Expression of neutrophil-related genes in human samples.

	CXCR2	CXCR1	IL17A	NOS2	MPO	CXCL3	CXCL5	CXCL8	CXCL6	ELANE	CTSG	CXCL2	CXCL7	CXCL1
MEAN(R)	208.089	197.244	87.4742	98.7889	16.2153	50.5724	19.3574	54.4989	7.56835	38.0585	136.787	8.87476	119.519	22.3482
STANDARD DEVIATION	69.761	96.442	30.401	67.474	8.205	25.416	7.473	46.34	2.208	14.042	40.764	3.148	247.595	32.185
MEAN(S)	136.445	115.511	85.8337	81.5115	8.73933	36.6042	13.4168	39.9076	9.19468	30.0681	146.353	11.9816	54.1024	14.949
STANDARD DEVIATION	19.809	41.735	18.228	49.583	3.625	14.686	3.291	51.389	3.312	16.513	40.134	7.798	98.898	17.365
STUDENT T TEST	0.00625	0.02477	0.88545	0.517	0.01692	0.14908	0.03437	0.49398	0.18111	0.23465	0.58968	0.21119	0.45655	0.53308

**Table 2:** Primers used in this study.

Gene Name	Forward (5′–3′)	Reverse (3′–5′)
***Il-23***	GCTGTGCCTAGGAGTAGCAG	TGGCTGTTGTCCTTGAGTCC
***Gapdh***	AGCCTCGTCCCGTAGACAAAA	GATGACAAGCTTCCCATTCTCG
***Il-1β***	GCAACTGTTCCTGAACTCAACT	ATCTTTTGGGGTCCGTCAACT
***Ccl4***	TGACCAAAAGAGGCAGACAG	CTCCCCCAAAAAAACAAAAC
***Ccl3***	TTCTCTGTACCATGACACTCTGC	CGTGGAATCTTCCGGCTGTAG
***Ccl2***	TTAAAAACCTGGATCGGAACCAA	GCATTAGCTTCAGATTTACGGGT
***Inos***	GTTCTCAGCCCAACAATACAAGA	GTGGACGGGTCGATGTCAC

**Table 3:** Antibodies used in this study.

Protein	Primary Antibody	Source	Application	Dilution
PCNA	anti-PCNA	Santa Cruz, TX, USA Cell Signaling, MA, USA	WB IF	1:1000 1:200
Human neutrophil	Anti-Myeloperoxidase	Abcam, MA,	IHC	1:100
GAPDH	Anti GAPDH: sc-53;02	Santa Cruz, TX	WB	1:2000
Anti-Histone H3	(citrulline R2 + R8 + R17): ab5103	Abcam, MA,	IF	1:200

## Supplementary Materials

The following are available online at https://www.mdpi.com/2072-6694/12/10/2884/s1, Figure S1: the whole blot (uncropped blots) showing.
