# Supplementary figures and images for: Breast Cancer Cell–Neutrophil Interactions Enhance Neutrophil Survival and Pro-Tumorigenic Activities
Source: Cancers (Basel). 2020 Oct 8;12(10):2884. doi: 10.3390/cancers12102884 (PMC7599756; doi:10.3390/cancers12102884)

# MPRO

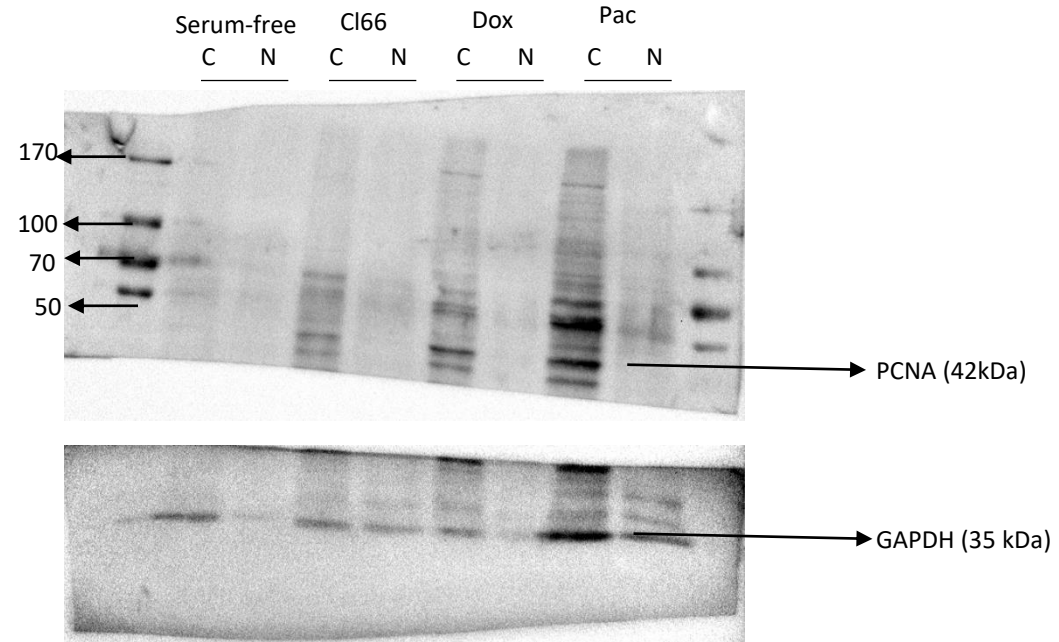

Supplement: Supplementary file 1 [file cancers-12-02884-s001.pdf]
